# Engineering skin microphysiological systems for transdermal drug screening based on strategic model selection and quantitative prediction roadmaps

**DOI:** 10.1016/j.mtbio.2026.103223

**Published:** 2026-05-15

**Authors:** Jongwoo Ahn, Geonho Jin, Minji Cho, Minho Do, Jungho Ahn, Jihoon Ko, Seokyoung Bang

**Affiliations:** a Department of Biomedical Engineering, Dongguk University, Goyang, 10326, Republic of Korea; b Department of MetaBioHealth, Sungkyunkwan University, Suwon, 16419, Republic of Korea; c Department of Biophysics, Institute of Quantum Biophysics, Sungkyunkwan University, Suwon, 16419, Republic of Korea; d Department of BioNano Technology, Gachon University, Seongnam-si, Gyeonggi-do, 13120, Republic of Korea

**Keywords:** Transdermal drug delivery systems, Skin microphysiological systems, Vascularization, Physiological fluidic conditions, *In vitro*-*in vivo* extrapolation

## Abstract

As transdermal drug delivery systems (TDDS) gain increasing importance, the need for screening platforms that accurately recapitulate human skin physiology has become essential. Conventional static evaluation methods, such as Franz diffusion cells, remain limited in their ability to recapitulate *in vivo* dynamic transport, metabolism, and clearance processes. This review examines the technical significance and emerging applicability of skin microphysiological systems (MPS) as next-generation platforms for transdermal drug screening. We first outline the multilayered structure of the skin, principal permeation pathways, and key physiological determinants governing transdermal absorption, while critically assessing the limitations of conventional *ex vivo* and *in vitro* methods. We then systematically present the design principles, evaluation metrics, and representative applications of diverse skin MPS, classified by vascularization and flow conditions. Through this analysis, we highlight how skin MPS improve the accuracy of permeability assessment and enable more physiologically relevant evaluation of drug efficacy and toxicity. Importantly, we emphasize that the primary value of skin MPS lies not in maximal complexity, but in the strategic selection of model architecture tailored to specific research objectives. Finally, we discuss future directions toward quantitative prediction, including integration of skin MPS data with physiologically based pharmacokinetic (PBPK) models via *in vitro*–*in vivo* extrapolation (IVIVE), alongside key challenges related to structural fidelity, standardization, and regulatory translation.

## Graphical abstract

**Figure:**
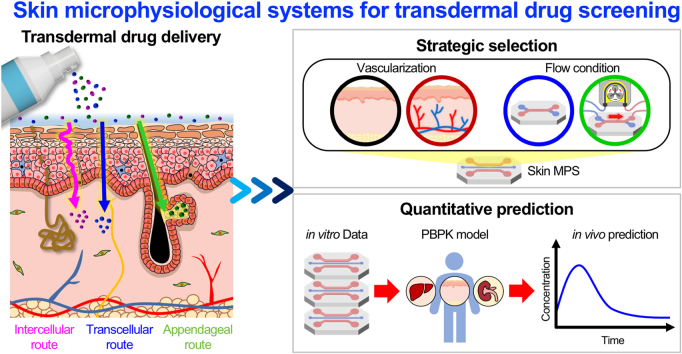

## 1 Introduction

The skin is the largest organ of the human body, acting as the primary barrier that protects the body from external environment. It also performs various physiological functions such as thermoregulation, moisture retention, and sensory perception [1]. Its highly organized multilayered architecture, composed of the epidermis, dermis, and hypodermis, exhibits a dual characteristic—it blocks external stimuli and harmful substances while allowing selective absorption of certain molecules into the body [2]. Owing to these unique properties, the skin has been recognized as an important non-invasive route for drug delivery [3,4]. Transdermal absorption describes the physiological process by which drugs or chemical substances transverse the stratum corneum and subsequently penetrate the viable epidermal and dermal layers. This process is regulated by both the structural characteristics of the skin and the physicochemical properties of the permeant [5].

Transdermal drug delivery systems (TDDS) exploits the selective permeability of the skin to deliver therapeutic agents in a noninvasive, sustained, and controlled manner [6]. Compared with oral administration, TDDS can bypass hepatic first-pass metabolism, maintain more stable plasma drug concentration, enhance therapeutic efficacy, and reduce systemic side effects [7]. In recent years, TDDS applications have expanded beyond small molecules such as nicotine and hormonal agents to include proteins, peptides, and nanoparticle-based formulations. In parallel, TDDS has gained increasing attention for its potential in personalized pharmacotherapy and noninvasive physiological monitoring [8,9]. Consequently, TDDS has become a key focus of research across the pharmaceutical, medical, and cosmetic industries [10].

The overarching objective of transdermal drug delivery research is to ensure that therapeutics efficiently cross the skin barrier and reach systemic circulation at predictable rates [4]. However, transdermal absorption exhibits substantial variability across individuals, anatomical sites, and skin conditions, making accurate prediction fundamentally challenging using conventional evaluation models [11]. Animal models often fail to recapitulate human transdermal permeation due to interspecies differences in skin structure and physiology, while two-dimensional (2D) cell culture models lack the complexity required to recapitulate the stratified barrier, vascular clearance, and metabolic responses of human skin [12]. Likewise, Franz diffusion cells and *ex vivo* excised skin models primarily assess passive diffusion driven by concentration gradients under static conditions. They lack the ability to reproduce physiological factors such as perfusion-driven clearance and metabolic activity [13]. Consequently, although these traditional approaches remain valuable for barrier permeability analysis, they are insufficient to support quantitative *in vitro–in vivo* extrapolation (IVIVE), particularly with regard to predicting systemic exposure following transdermal drug administration [14]. To address these limitations, vascularized skin microphysiological systems (MPS) incorporating perfusable vascular components have emerged as next-generation platforms for TDDS research [15].

This review provides an overview of the physiological structure of the skin and the mechanisms underlying transdermal absorption, and critically evaluates the limitations of conventional *in vivo, ex vivo,* and *in vitro* approaches. We then introduce a classification framework for skin MPS based on the presence or absence of vascularization and the flow condition (static versus dynamic conditions). Representative TDDS studies employing skin MPS are systemically organized according to this framework, highlighting how different design strategies have been applied to evaluate drug permeability and simulate systemic exposure. Finally, we discuss future directions for improving reproducibility, developing patient-derived models, expanding toward multi-organ MPS integration, and enabling industrial translation, highlighting the potential of skin MPS to serve as standardized platforms for next-generation TDDS.

## 2 Transdermal absorption mechanisms

Transdermal absorption is a complex physiological process where drugs or exogenous substances are transported into the body across the skin barrier. Owing to its relevance to drug delivery, cosmetic formulation, and toxicity assessment, this process has attracted increasing attention across diverse biomedical and industrial fields [16,17]. Transdermal absorption is governed by the structure of the skin, the route of penetration, and the physicochemical properties of the permeant [6]. Accordingly, mechanistic understanding of these determinants is essential for predicting penetration behavior and rationally designing effective TDDS.

**Fig. 1:**
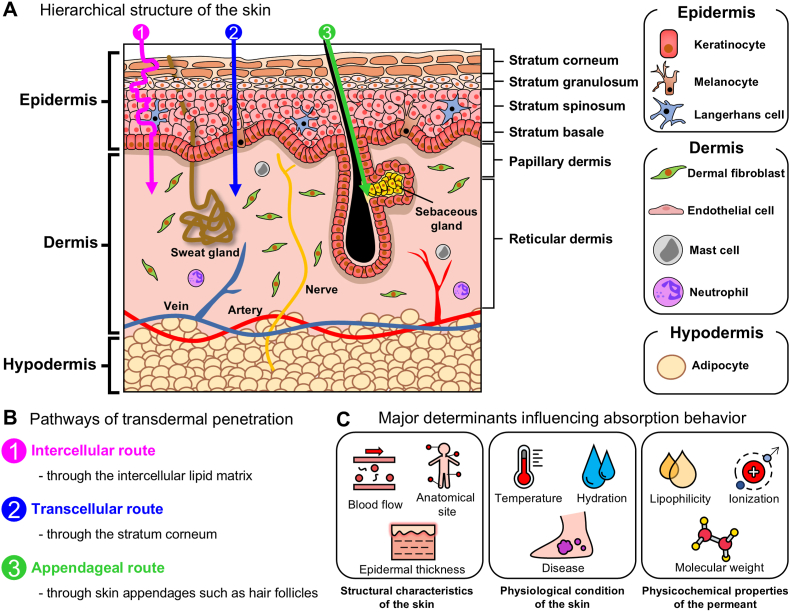
**Overview of transdermal absorption mechanisms and key determinants.** (A) Schematic of human skin architecture, including the epidermis (stratum corneum, stratum granulosum, stratum spinosum, and stratum basale), dermis (papillary and reticular dermis), and hypodermis, along with major skin appendages and representative cell types in each layer. (B) Pathways of transdermal penetration: (1) intercellular route through the intercellular lipid matrix of the stratum corneum, (2) transcellular route through the stratum corneum and viable epidermis, and (3) appendageal route through skin appendages such as hair follicles and glands. (C) Major determinants influencing absorption behavior, categorized into structural characteristics of the skin (blood flow, anatomical site, and epidermal thickness), physiological condition of the skin (temperature, hydration, and disease state), and physicochemical properties of the permeant (lipophilicity, ionization, and molecular weight).

In this section, we describe the fundamental mechanisms of transdermal absorption by focusing on three key aspects: (i) the hierarchical structure of the skin, (ii) the principal pathways of transdermal penetration, and (iii) the major determinants influencing absorption behavior (Fig. 1). In addition, we briefly summarize conventional experimental approaches and evaluation methods that have been used to investigate these mechanisms, providing context for the limitations addressed by emerging skin MPS.

### 2.1 The hierarchical structure of the skin

To understand the principles of TDDS and the mechanisms by which drugs traverse the skin barrier, it is essential to first outline the skin architecture. The skin consists of three primary layers—the epidermis, dermis, and hypodermis—whose distinct structural and functional properties collectively determine barrier function as well as the route and rate of transdermal drug absorption [18,19].

#### 2.1.1 Epidermis

The epidermis is the outermost layer of the skin and plays a central role in protecting the body from external environments while maintaining hydration [20]. It is composed predominantly of keratinocytes, along with specialized cells such as melanocytes and Langerhans cells [21]. As the epidermis lacks blood vessels, nutrients and oxygen are supplied by diffusion from the underlying dermis [1]. Epidermal keratinocytes undergo continuous proliferation and differentiation, giving rise to four distinct sublayers: the stratum basale, stratum spinosum, stratum granulosum, and the outermost stratum corneum [22].

The stratum basale is the proliferative layer in which keratinocytes actively divide and initiate upward migration during differentiation [23]. Above this, the stratum spinosum is characterized by prominent intercellular junctions that provide mechanical integrity to the epidermis [24], and it also contains Langerhans cells that contribute to immune surveillance against external pathogens [25]. In the stratum granulosum, keratinocytes accumulate keratin and lipid-rich lamellar bodies, marking the onset of functional barrier formation [24]. The outermost stratum corneum consists of flattened, enucleated corneocytes embedded within an ordered lipid matrix [26]. This layer constitutes the primary barrier to transdermal transport and represents the dominant diffusion barrier governing the permeation of most substances across the skin [26]. The epidermis maintains skin homeostasis through continuous self-renewal, balancing cell proliferation with terminal differentiation and desquamation [20].

#### 2.1.2 Dermis

The dermis is a thick connective tissue layer located beneath the epidermis and serves as the principal contributor to the skin's structural integrity and physiological function [27]. It consists of an extracellular matrix (ECM) rich in collagen and elastin fibers, together with diverse cell populations including fibroblasts, immune cells, and vascular endothelial cells. These components collectively support skin elasticity, mechanical strength, wound healing, thermoregulation, and nutrient supply [28]. The dermis is commonly subdivided into the papillary dermis and the reticular dermis. The papillary dermis lies directly beneath the epidermis and contains dense microvascular networks and sensory nerve endings, facilitating metabolic exchange and sensory function. In contrast, the reticular dermis is thicker and contains coarser fibrous structures, larger blood vessels, sweat glands, sebaceous glands, and hair follicles [29]. From a transdermal delivery perspective, the dermis represents the first vascularized compartment encountered by substances that successfully penetrate the epidermis barrier. As such, it plays a critical role in determining local drug distribution, clearance into systemic circulation, and the initiation of biological responses following skin penetration [12].

#### 2.1.3 Hypodermis

The hypodermis, located beneath the dermis, is a connective tissue layer enriched in adipose tissue that anchors the skin to underlying muscles and internal organs [30]. It is primarily composed of adipocytes that store neutral lipids, along with fibrous components, and contains blood vessels, nerve fibers, and immune cells [31]. Functionally, the hypodermis contributes to thermal insulation, mechanical shock absorption, and energy storage, while also serving as a conduit for vascular and neural networks supplying the overlying skin layers [30]. In addition to its structural roles, adipose tissue in the hypodermis secretes metabolic hormones such as leptin and adiponectin, thereby influencing systemic metabolic regulation [32]. As the terminal layer in the transdermal absorption pathway, the hypodermis acts as the final interface through which absorbed compounds pass before entering systemic circulation [33]. Consequently, its composition and vascular connectivity can influence the downstream distribution and pharmacokinetics of transdermally delivered drugs.

### 2.2 The principal pathways of transdermal penetration

Transdermal absorption refers to the physiological process by which substances applied to the skin surface penetrate the stratum corneum and underlying epidermal and dermal layers before ultimately reaching systemic circulation [6]. This process plays a critical role in a wide range of applications, including drug delivery, toxicity assessment, and cosmetic development [12]. Although the skin exhibits a robust barrier function, certain molecules are capable of permeating the stratum corneum or entering the skin via appendageal structures such as hair follicles and sweat glands [6]. Through these mechanisms, absorbed substances may exert either localized effects within the skin or systemic effects following vascular uptake.

Transdermal absorption generally occurs through three major pathways [34]. The intercellular route is the predominant pathway, in which substances diffuse through the lipid matrix occupying the intercellular spaces between corneocytes in the stratum corneum [35]. Because this matrix is highly lipophilic, compounds with lipophilic characteristics, such as nicotine and vitamin E, preferentially permeate via this route [8]. The transcellular route involves the direct passage of substances across corneocytes and keratinocytes [6]. Hydrophilic compounds, including fluorouracil, may utilize this pathway; however, repeated partitioning across alternating lipid and protein domains renders transcellular transport generally less favorable than intercellular diffusion [36]. The appendageal route allows substances to bypass portions of the stratum corneum by penetrating through skin appendages such as sweat glands, sebaceous glands, and hair follicles [37]. Although appendages occupy only a small fraction of the total skin surface area, this pathway can contribute to the permeation of certain molecules, including ionized compounds and particulate or nanostructured formulations [38].

Following penetration of the stratum corneum via these pathways, drugs diffuse through the viable epidermis and dermis and are subsequently taken up by the dermal capillary network, enabling entry into systemic circulation [39]. Accordingly, the objective of TDDS is to achieve therapeutically relevant blood concentrations through controlled skin permeation while circumventing first-pass hepatic metabolism [7]. For an *in vitro* evaluation perspective, accurately capturing this process requires models that incorporate a perfused vascular compartment. Such systems enable more physiologically relevant assessment of drug permeation and clearance by mimicking vascular uptake after barrier traversal, thereby providing a critical foundation for quantitative pharmacokinetic prediction using physiologically based pharmacokinetic (PBPK) modeling frameworks [4].

### 2.3 The major determinants influencing absorption behavior

Transdermal absorption occurs through the interplay of multiple factors, resulting in substantial interindividual and site-specific variability in absorption rates [40]. In general, transdermal absorption is primarily influenced by three major determinants: (i) structural characteristics of the skin, (ii) physicochemical condition of the skin, and (iii) physicochemical properties of the permeant.

#### 2.3.1 Structural characteristics of the skin

The structural features of the skin, including blood flow, sebaceous gland density, and stratum corneum thickness, vary across anatomical sites and exert a strong influence on transdermal permeability [41]. Regions such as the eyelids, genital area, and forehead, which are characterized by a thin stratum corneum and high density of sebaceous glands, exhibit relatively high absorption rates. In contrast, areas with thick and highly keratinized skin, such as the palms and soles, show markedly reduced permeability [1]. Quantitatively, drug absorption through genital skin has been reported to be approximately 40-fold higher than that through the inner forearm, whereas absorption through the palm is reduced to roughly one-tenth of that level [42].

Beyond thickness alone, the composition, quantity, and spatial organization of lipids within the stratum corneum critically regulate the transport of lipophilic molecules [43]. Because diffusion through the intercellular lipid pathway is tortuous, alterations in lipid composition or lamellar organization due to anatomical variation, disease, or formulation effects can significantly modulate permeability [44,45]. Variations in appendages density, size, and distribution across body sites therefore contribute to differences in absorption efficiency and early drug uptake kinetics [6].

#### 2.3.2 Physiological condition of the skin

In addition to structural features, transdermal absorption is strongly influenced by physiological skin conditions that vary among individuals and over time [46]. Even when the identical formulations are applied at the same concentration, absorption rates may differ depending on parameters such as skin hydration, temperature, inflammatory status, and disease state [47,48]. Because these factors can change dynamically, they represent important sources of variability that must be considered in TDDS design and evaluation.

Skin hydration is one of the most direct regulators of barrier permeability [49]. Increased hydration disrupts the ordered lipid structure of the stratum corneum, expanding intercellular diffusion pathways and thereby enhancing permeability [50]. The principle underlies the use of occlusion—such as films or patches—that reduce transepidermal water loss and promote local hydration to facilitate drug penetration [51].

Skin temperature also affects transdermal absorption by modulating both diffusion kinetics and dermal blood perfusion [52]. Elevated temperatures increase molecular mobility, accelerating diffusion across the skin barrier, while vasodilation enhances clearance of absorbed substances into systemic circulation [53,54]. Moreover, inflammatory conditions such as dermatitis or atopic skin disorders can compromise barrier integrity, resulting in abnormal or excessive drug absorption [55].

Overall, these physiological conditions play a crucial role in determining both the extent and route of transdermal absorption. Quantitative evaluation of skin condition-dependent effects is therefore essential for ensuring the safety, reproducibility, and predictive accuracy of TDDS assessments.

#### 2.3.3 Physicochemical properties of the permeant

Transdermal absorption is strongly influenced by the intrinsic physicochemical characteristics of the permeant [56]. Key parameters such as molecular weight, lipophilicity, concentration gradient, ionization state, and solubility determine whether a compound can traverse the skin barrier and at what rate [57]. Among these, molecular weight is a major determinant of skin permeability [58]. In general, compounds with molecular weights below approximately 500 Da can penetrate the stratum corneum more effectively, whereas permeability decreases sharply as molecular size increases [[58], [59], [60]].

Lipophilicity, typically expressed as the octanol–water partition coefficient (log P), governs a compound's ability to partition into both the lipid-rich stratum corneum and the aqueous environments of the viable epidermis and dermis [61]. Optimal transdermal absorption is typically observed for compounds with log P values between 1 and 3 [62,63]. Compounds with low log P exhibit insufficient lipid affinity to cross the stratum corneum, whereas excessively high log P values lead to retention within lipid domains, limiting diffusion into deeper layers. As a result, amphiphilic molecules with balanced hydrophilic and lipophilic properties demonstrate the most favorable absorption behavior [64].

The concentration gradient across the skin acts as the primary driving force for diffusion [42]. According to Fick's law, higher drug concentrations at the skin surface led to increased permeation rates, forming the theoretical basis for maintaining sustained concentration profiles in TDDS designs [65]. In addition, the ionization state of a compound plays an important role. Because the skin surface typically exhibits a mildly acidic pH (approximately 4–6), non-ionized species—which possess greater lipid solubility—penetrate the stratum corneum more efficiently [66,67]. Formulation characteristics further influence transdermal absorption by determining drug solubility, stability, release kinetics, and availability at the skin surface [34,68,69]. For effective permeation, the drug must be molecularly dissolved within the formulation, as undissolved particles cannot directly diffuse through the skin [70]. To accomplish this, compounds must exhibit sufficient solubility in both the lipid-rich stratum corneum and the aqueous environments of the deeper skin layers [71].

Overall, the physicochemical behavior of the permeant reflects complex interactions among multiple factors rather than dependence on a single variable. Comprehensive consideration of these parameters is therefore essential for rational TDDS design [62].

### 2.4 Conventional models and methodologies for evaluating transdermal drug delivery systems

Conventional approaches for evaluating transdermal absorption can be broadly categorized into *in vivo*, *ex vivo*, and *in vitro* methods (Table 1). Each approach offers distinct advantages and limitations in terms of physiological relevance, quantitative accuracy, reproducibility, and regulatory acceptance.

**Table 1:** Comparison of *in vivo*, *ex vivo*, *in vitro* and skin MPS models for evaluating transdermal drug delivery systems.

Model type	Evaluation Model	Advantages	Limitations	Ref.
***In vivo***	• Human tape-stripping for dermatopharmacokinetic (DPK) • Animal mass balance test (OECD TG 427)	• Physiological relevance under *in vivo* conditions • Systemic pharmacokinetic (PK) profiling	• Invasive sampling and the need for recovery-efficiency corrections • Interspecies differences in permeability • Ethical constraints and high inter-individual variability	[12,[72], [73], [74]]
***Ex vivo***	• Excised human or animal skin mounted on vertical diffusion cell (Franz or flow-through type) • OECD TG 428	• Preservation of native skin architecture including the stratum corneum • Quantitative permeation metrics (steady-state flux (Jss) and permeability coefficient (Kp)) • Franz: simple design, cost-effectiveness, and suitability for low-permeability compounds • Flow-through: improved maintenance of sink conditions and compatibility with automated sampling	• Limited recapitulation of perfusion, systemic metabolism, and appendage-mediated transport contributions • Variability due to skin source, thickness, and pre-treatment • Franz: difficulty in maintaining sink conditions and requirement for manual sampling • Flow-through: Analyte dilution, necessitating high-sensitivity analysis and high initial setup costs	[13,15,73,[75], [76], [77], [78], [79], [80], [81]]
***In vitro***	• 2D monolayer cell culture and Transwell-based systems • Synthetic membrane/RhE-based diffusion cell	• Convenient TEER measurement and formulation comparison • High standardization and reproducibility • Early-stage screening and relative permeability ranking across compounds	• Limited physiological relevance (stratum corneum, perfusion, metabolism) • Underestimation of barrier function • Requirement for cross-validation with other methods	[73,82,83]
**Skin MPS**	• Skin MPS	• Recapitulation of perfusion and barrier interactions • Long-term culture and real-time monitoring • Emerging New Approach Methodologies (NAMs) potential	• Lack of standardized and validated protocols • Complex fabrication and technical operational demands	[15,84,85]

In *in vivo* evaluation of transdermal absorption typically relies on minimally invasive pharmacokinetic measurements in humans or mass balance studies in animal models [12]. A representative human-based method is the tape-stripping dermatopharmacokinetic (DPK) technique, in which the stratum corneum is sequentially removed using adhesive tapes at defined time points following topical application. Drug concentration profiles across the stratum corneum are then analyzed to estimate penetration depth and absorption kinetics [72]. In animal studies, the OECD Test Guideline (TG) 427 recommends the application of a finite dose of the test substance while preventing evaporation and ingestion, followed by quantitative analysis of skin, blood, urine, and feces to determine the absorbed dose, skin retention, and overall recovery [73,86]. Despite their physiological relevance, *in vivo* approaches suffer from inherent limitations in quantitativeness and reproducibility. Direct measurements are largely restricted to the stratum corneum, recovery corrections are required, and results are strongly influenced by interspecies differences in skin structure and permeability, as well as ethical and practical constraints [12,74].

*Ex vivo* assessment of transdermal absorption is most commonly performed using vertical diffusion cell system, in which excised skin is mounted between donor and receptor compartments. Key quantitative parameters obtained from these experiments include cumulative permeation, steady-state flux (J_ss_), lag time, permeability coefficient (K_p_), and recovery rate [15]. Vertical diffusion cells are generally classified into static systems, commonly referred to as Franz diffusion cells, and flow-through systems [13], both of which are accepted under OECD TG 428 [75,76]. Franz cells are widely used due to their simplicity, low cost, but they are limited by sink-condition maintenance and manual sampling variability [15,73]. Flow-through diffusion cells address some of these limitations by enabling continuous medium replacement, automated sampling and improved experimental efficiency [77,78]. However, despite their utility, vertical diffusion cells remain limited in their ability to reproduce key physiological features of *in vivo* transdermal absorption, including vascular perfusion, metabolic activity, and appendage-mediated transport pathways [13].

Traditional *in vitro* models for screening transdermal absorption include 2D monolayer cell cultures, transwell-based systems, and membrane permeation assays using vertical diffusion cells. In this context, vertical diffusion cell are often equipped with reconstructed human epidermis (RhE) models or synthetic membranes instead of excised skin [87]. These systems are convenient for formulation screening, barrier integrity assessments, and transepithelial electrical resistance (TEER) measurements, and they offer high reproducibility and experimental standardization suitable for early-stage screening [82]. However, conventional *in vitro* models frequently underestimate barrier function due to the incomplete development of a fully stratified corneum. Moreover, they lack critical physiological features such as active metabolism, vascular perfusion, and follicular or glandular transport, limiting their ability to predict system exposure [73,83]. As a result, data obtained from these models often require cross-validation with *ex vivo* or *in vivo* approaches.

Unlike conventional *in vitro* assays, skin MPS platforms are emerging as non-guideline New Approach Methodologies (NAMs) designed to overcome many of the aforementioned limitations. By integrating microfluidic perfusion and vascular components, and controlled tissue architectures, skin MPS enable dynamic reproduction of sink conditions, metabolic activity, and barrier–vascular interactions in a stepwise and physiologically relevant manner [15,84,85]. These capabilities position skin MPS as promising platforms for bridging conventional transdermal screening with quantitative, mechanism-informed prediction through IVIVE.

## 3 Skin MPS

MPS have emerged as next-generation *in vitro* platforms designed to recapitulate key aspects of the human tissue microenvironment, enabling applications in drug screening, toxicity assessment, and disease modeling [88,89]. To address the limitations of conventional *in vitro* approaches, micro-engineered skin models, collectively referred to as skin MPS, have been actively developed to recapitulate human skin physiology under more realistic and dynamic conditions [90].

Two primary strategies have been adopted for constructing skin MPS: top-down and bottom-up approaches [91,92]. The top-down approach involves harvesting intact human skin, typically using a biopsy punch, and integrating the excised tissue directly into a microfluidic device. In representative implementations, native skin samples are mounted onto a porous membrane, with culture medium supplied through a lower microchannel while the epidermal surface is maintained under air-liquid interface (ALI) conditions [91,93]. This configuration preserves the original stratum corneum barrier and native appendages, providing strong barrier integrity and enabling the establishment of baseline transdermal absorption profiles using human skin. However, top-down skin MPS are constrained by the limited viable culture duration of *ex vivo* tissue and the restricted ability to independently control cellular composition, genetic background, or tissue remodeling [94,95]. Moreover, due to the difficulty of reconstructing or reperfusing the complex native vascular network within a microfluidic device, top-down approaches are predominantly applied to non-vascularized skin MPS. In contrast, the bottom-up approach reconstructs skin tissue by assembling its constituent cellular components within engineered matrices. Typically, dermal fibroblasts are embedded within hydrogels such as collagen to form a dermal compartment, followed by the sequential introduction of epidermal keratinocytes and, in some cases, vascular endothelial cells. The constructs are subsequently transitioned to ALI culture to induce epidermal stratification and keratinization [96]. This method enables precise control over cellular composition, ECM density, and microenvironmental cues, making it particularly suitable for disease modeling and mechanistic studies. However, the resulting stratum corneum may not fully achieve the structural and functional maturity observed in native human skin and the faithful reconstruction of complex appendages such as hair follicles, sweat glands, and neural networks remains challenging [97].

**Fig. 2:**
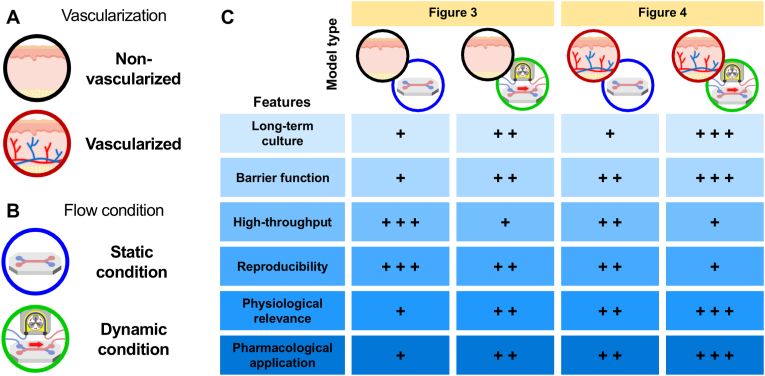
**Classification framework for microfluidic skin MPS based on vascularization and flow conditions.** (A) Schematic categorization of skin MPS into non-vascularized and vascularized models according to the presence of a perfusable vascular layer. (B) Flow conditions used in skin MPS, classified as static (no continuous perfusion) or dynamic (perfusion-driven microfluidic environment). (C) Matrix summarizing representative combinations of model types and their relative advantages across key features relevant to TDDS research, including long-term culture, barrier function, high-throughput compatibility, reproducibility, physiological relevance, and pharmacological application. The "+" symbol indicates a qualitative, relative ranking ("+++" high, "++" moderate, and "+" low).

In the following sections, we systematically review skin MPS platforms with a focus on their relevance to transdermal compound screening. We first describe the general principles of static and dynamic conditions in MPS. The presence or absence of a vascular layer—representing the ultimate sink for transdermally absorbed drugs—is established as the primary classification criterion. Within each category, models are further distinguished based on the nature of fluidic conditions, specifically static versus dynamic perfusion environments (Fig. 2).

### 3.1 Static and dynamic condition

Human tissues are continuously exposed to dynamic microfluidic environments generated by blood flow and interstitial fluid movement [98,99]. These flow-driven conditions play a critical role in regulating cellular viability, barrier integrity, and drug transport behavior [100]. Accordingly, MPS can be broadly classified into static conditions, in which fluid flow is absent, and dynamic conditions, which incorporate hydrodynamic force through controlled fluid circulations.

Under static conditions, no active fluid flow is present, and drugs or external substances are transported primarily by passive diffusion driven by the concentration gradient [90]. Static platforms offer practical advantages, including simple fabrication, straightforward operation, and compatibility with high-throughput screening formats [101,102]. However, the lack of fluid exchange limits nutrient replenishment and waste removal, often resulting in metabolite accumulation, uneven compound distribution, and local depletion of oxygen and nutrients. As a result, although static models are well suited for short-term permeability screening and basic barrier function assessment, prolonged culture under static conditions can compromise cellular functionality and physiological relevance [103].

On the other hand, dynamic conditions involve continuous or intermittent fluid circulation generated by pumps, gravity-driven flow, or capillary flow [104]. The resulting laminar flow produces defined levels of shear stress on cells, a hallmark of microfluidic systems. Shear stress has been shown to modulate cell morphology, alignment, and intracellular signaling, particularly in vascular endothelial cells, which adopt *in vivo*-like elongated morphologies and enhanced junctional organization under flow [105]. These effects promote the formation of tight junctions and strengthen barrier integrity [106]. In addition, dynamic perfusion alleviates diffusion limitations inherent to static system by enabling continuous nutrient and oxygen supply while effectively removing metabolic byproducts, thereby supporting long-term cell viability and functional stability [107].

### 3.2 Non-vascularized skin MPS

#### 3.2.1 Static condition

Non-vascularized skin MPS are defined as skin models that lack perfusable blood vessels and are primarily composed of epidermal and dermal components [93,97,108]. Under static conditions, nutrients and oxygen are supplied mainly through diffusion from the underlying microfluidic channel, while the upper surface of the tissue is maintained under ALI conditions to promote epidermal differentiation and keratinization [97]. Even in the absence of vascularization and active perfusion, top-down skin MPS incorporating *ex vivo* human skin microtissues can be constructed. These models preserve native tissue architecture and enable functional readouts of innate immune responses and drug effects without the need for vascular components or fluid flow [93]. Furthermore, certain non-vascularized static skin MPS have demonstrated the ability to reproduce complex skin-associated structures, such as nerve-epidermal interfaces, that closely resemble *in vivo* organization [97]. From a practical standpoint, non-vascularized static skin MPS offer several advantages. Their simple system design and absence of flow-related variables facilitate ease of operation, enhanced reproducibility and experimental standardization. These features make such platforms particularly well suited for early-stage screening, baseline permeability assessment, and comparative evaluation of formulations [108]. However, the lack of vascular perfusion and limited fluid exchange restrict the faithful reproduction of metabolic activity, immune cell crosstalk, and dynamic drug clearance. As a result, the applicability of these models for long-term culture and quantitative analysis of systemic drug responses remains constrained [95,103].

#### 3.2.2 Dynamic condition

Non-vascularized dynamic skin MPS incorporate controlled fluid flow to supply nutrients and oxygen to skin tissues and to remove metabolites, despite the absence of a perfusable vascular component [[109], [110], [111], [112], [113], [114], [115], [116], [117], [118], [119], [120], [121], [122], [123]]. Similar to static configurations, the upper compartment of the device is maintained ALI conditions to promote epidermal stratification and keratinization. In contrast to static models, continuous perfusion through the lower microchannel generates a more physiologically relevant microenvironment by supplying nutrients, oxygen, and soluble factors that support keratinocyte differentiation and dermal fibroblast-mediated ECM deposition. Concurrently, flow-induced shear stress modulates cellular mechanotransduction, leading to increased expression of tight junction and barrier-associated proteins. These effects are reflected in elevated TEER values [117], and a reduced permeation of model compounds such as fluorescein isothiocyanate (FITC)-dextran [113].

Fluid flow in dynamic skin MPS can be generated using various approaches, including external pumps, gravity-driven flow, or capillary-driven flow [107,111]. The introduction of dynamic perfusion expands the range of experimental applications. Controlled shear stress supports long-term cell viability and tissue maturation, making non-vascularized dynamic skin MPS suitable for evaluating transdermal absorption kinetics under sustained culture conditions [120]. In addition, these platforms enable the modeling of pathophysiological skin responses, such as irritation, inflammation, and toxicity, through exposure to external stimuli including sodium lauryl sulfate (SLS) or *Propionibacterium acnes* [121]. More advanced implementations have integrated auxiliary modules, such as hormone secretion units, in which estradiol and testosterone are delivered through the Christmas tree-shaped microfluidic channels to generate individual or mixed gradient. These configurations allow the investigation of sex-specific skin responses, providing a foundation for personalized drug and cosmetic testing [123]. Despite these advantages, non-vascularized dynamic skin MPS cannot replicate key vascular functions, such as endothelial-mediated uptake, active clearance, and vascular signaling. Consequently, their ability to quantitatively predict systemic drug exposure remains constrained [95].

### 3.3 Vascularized skin MPS

#### 3.3.1 Static condition

Vascularized static skin MPS incorporate vascular endothelial cells together with skin-resident cells to establish a coupled skin–vascular barrier architecture, while relying exclusively on diffusion-based transport in the absence of active fluid flow [124]. Compared with non-vascularized models that reconstruct only the epidermal and dermal compartments, the inclusion of a vascular interface enhances physiological relevance by introducing an endothelial barrier that serves as the initial gateway for systemic uptake. At the same time, these platforms retain the structural simplicity and operational accessibility characteristic of static systems. Unlike non-vascularized skin MPS, which may be constructed using either top-down or bottom-up strategies, vascularized skin MPS are generated exclusively through bottom-up approaches. In typical configurations, vascular endothelial cells are cultured along the lower surface of a dermis-mimicking ECM hydrogel, creating an endothelial barrier beneath the reconstructed skin equivalent. This configuration enables the formation of a spatially organized skin-vascular interface while preserving diffusion-dominated transport across both tissue compartments. Despite the absence of perfusion, the presence of an endothelial layer allows investigation of biological processes that cannot be captured in non-vascularized systems. For example, cytokine-driven immune cell migration across epithelial and endothelial barriers can be observed and analyzed through transepithelial and transendothelial transport mechanisms [124]. However, because molecular transport and cell signaling in these systems remain governed by passive diffusion, vascularized static skin MPS are limited in their ability to reproduce flow-dependent phenomena such as shear-responsive endothelial function, perfusion-mediated clearance, and dynamic pharmacokinetic behavior.

#### 3.3.2 Dynamic condition

Vascularized dynamic skin MPS integrate engineered skin tissue with a vascular component under flow conditions, thereby recreating a physiologically relevant microenvironment that most closely approximates native human skin physiology [92,[125], [126], [127], [128], [129], [130], [131], [132], [133], [134]]. Similar to non-vascularized dynamic models, the epidermal surface is maintained under ALI culture to induce keratinization, while controlled microfluidic flow is introduced through the lower channel to mimic blood circulation. In contrast to static vascularized systems, which provide only a diffusion-based endothelial barrier, dynamic skin MPS incorporate actively perfused vascular structure using multiple strategies. One approach establishes an endothelial barrier by seeding vascular endothelial cells along the lower surface of a membrane or hydrogel-based dermal compartment, enabling flow-mediated endothelial conditioning [128,132,134]. Alternatively, perfusable endothelial lumens can be formed directly within hydrogels using techniques such as 3D bioprinting or sacrificial templating with nylon wires, followed by endothelialization of the luminal surface to establish vascular channel [131,135].

Vascularized dynamic skin MPS support long-term culture and enhance epidermal barrier maturation by facilitating continuous delivery of oxygen and nutrients and efficient removal of metabolic waste through the vascular network. Shear stress applied to endothelial cells further enhances vascular barrier function and promotes phenotypic alignment with *in vivo* endothelium, contributing to a skin construct with increased physiological fidelity. As a result, these systems enable a broad range of applications. The presence of a perfused vascular layer allows the recruitment of immune cells into the skin construct and supports physiological processes such as leukocyte rolling, adhesion, and extravasation under flow, making the platform vascularized dynamic skin MPS particularly suitable for studying immune and inflammatory responses associated with infectious or inflammatory skin diseases [125,130].

More advanced implementations have extended vascularized skin MPS beyond the epidermal and dermal layers to include subcutaneous adipose tissue, reconstructed using 3D bioprinting together with perfusable vascular networks [135]. In such thick, metabolically active constructs, diffusion alone is insufficient to sustain tissue viability, as oxygen and nutrient gradients rapidly develop and adipocytes exhibit high metabolic demand. The integration of perfusable vascular channels mitigates hypoxia and nutrient deprivation in the central region of the tissue, thereby enabling stable long-term culture [136]. Furthermore, multi-organ MPS that interconnect skin with other organs such as the heart, liver, and bone through a shared vascular circulation allow integrated assessment of systemic PBPKs, inter-organ crosstalk, and tissue-specific toxicity within a single platform [128].

## 4 Skin MPS for the screening, evaluation, and quantification of transdermal drug delivery systems

As discussed in the preceding sections, skin MPS can be systemically categorized based on two key physiological parameters: (i) the presence or absence of a vascular layer and (ii) the use of static or dynamic fluidic conditions. Together, these parameters define the physiological scope of each platform and thus its suitability for specific transdermal drug screening objectives. Importantly, the value of skin MPS should, where possible, be interpreted in comparison with conventional methods rather than as a stand-alone demonstration of feasibility. In representative studies, skin MPS have provided more controlled evaluation of specific transport mechanisms than *in vivo* models and, in vascularized formats, offered the structural advantage of simultaneously evaluating topical and systemic exposure [133,137]. In addition, some skin MPS platforms have shown comparable or lower variability than Franz diffusion cells, supporting improved reproducibility in permeation testing [138]. These observations suggest that the main advantage of skin MPS lies not only in reproducing transdermal transport, but also in improving the physiological relevance, reproducibility, and translational interpretability of the resulting data. The following sections summarize representative case studies that employed skin MPS for TDDS screening and evaluation (Table 2, Table 3).

**Table 2:** Summary of skin MPS for evaluating transdermal drug delivery systems.

Vascularization	Flow condition	Ref	Biological Source	Dermal Matrix/Scaffold	Transdermal agent	Key results & implications
**Non-vascularized**	**Static**	Lee et al. [108]	(Cell-based) • Skin - Keratinocytes (primary) • Co-culture - Neurons (NSC-derived) - Hepatocytes (iPSC-derived)	Collagen type I	• Capsaicin • Lactic acid • Strontium chloride • Acetaminophen • Camphor	• Capsaicin and lactic acid increased neuronal Ca^2+^ signaling following transdermal exposure, which was attenuated by strontium chloride. • Acetaminophen and camphor induced glutathione depletion and increased reactive oxygen species (ROS) levels in the liver compartment. • Enabled simultaneous imaging-based quantification of sensory irritation and hepatotoxicity.
	**Dynamic**	Mohamadali et al. [91]	(Tissue-based) - *Ex vivo* human skin	Native human dermis	• Retinoic acid (RA)	• Quantified cumulative permeation and steady-state flux (Jss) through time-resolved effluent analysis. • Enabled long-term pumpless culture and quantification of transdermal diffusion parameters using intermittent flow induction.
		Lukacs et al. [139]	(Tissue-based) - *Ex vivo* mouse skin - *Ex vivo* rat skin	Native mouse and rat dermis	• Caffeine	• Topical application of caffeine increased cumulative permeation and steady-state flux (Jss) in tape-stripped skin, while permeation remained low in intact skin with a preserved barrier. • Enabled quantitative evaluation of transdermal drug absorption using minimal skin tissue and sample volumes.
		Bajza et al. [137]	(Tissue-based) - *Ex vivo* rat skin	Native rat dermis	• Quinidine • Erythromycin • PSC-833	• Treatment with the P-glycoprotein (P-gp) inhibitor PSC-833 reduced transdermal absorption of quinidine and erythromycin in *ex vivo* and *in vivo* models. • Enabled validation of P-gp–mediated directional transport and inhibition effects, demonstrating the applicability of dynamic skin MPS for skin transporter studies.
		Szederkenyi et al. [140]	(Tissue-based) - *Ex vivo* mouse skin - *Ex vivo* rat skin	Native mouse and rat dermis	• Caffeine • Dexamethasone • Diclofenac • Indomethacin • Piroxicam	• Caffeine permeation increased in psoriatic and allergic skin models, whereas in healthy skin piroxicam showed the highest and diclofenac the lowest permeation. • Dexamethasone and indomethacin exhibited biphasic kinetics, with an initial lag phase followed by accelerated absorption. • Enabled quantitative screening and prediction of transdermal drug delivery across barrier-impaired conditions and drug properties by integrating a three-compartment *in silico* model with skin MPS.
		De Mello et al. [141]	(Cell-based) • Skin - None • Co-culture - Cardiomyocytes (iPSC-derived) - Hepatocytes (primary)	Synthetic membrane (Polyethersulfone, Polyolefin)	• Diclofenac • Ketoconazole • Hydrocortisone • Acetaminophen	• Diclofenac, ketoconazole, hydrocortisone, and acetaminophen were evaluated in a heart-liver body MPS incorporating a Strat-M synthetic skin membrane. • Transdermal delivery led to attenuated cardiac and hepatic responses due to skin barrier resistance, whereas systemic administration induced clear dose-dependent effects. • Enabled simultaneous quantification of transdermal transport dynamics and organ-specific toxicity within a single recirculating multi-organ chip.
		Abaci et al. [107]	(Cell-based) • Skin - Keratinocytes (primary) - Dermal Fibroblasts (primary)	Collagen type I	• FAM-tagged oligonucleotides • Doxorubicin	• After topical application of FAM-tagged oligonucleotides, transport was quantified by fluorescence detection to determine the overall permeability coefficient (Kp). • Doxorubicin treatment reduced proliferation and induced tissue damage. • Enabled long-term pump-free maintenance of skin barrier integrity and differentiation for over three weeks, allowing quantitative analysis of drug permeability and toxicity.
		Alberti et al. [138]	(Cell-based) • Skin - Keratinocytes (N/TERT-1 cell line) - Dermal Fibroblasts (primary)	Fibrin	• Caffeine • Salicylic acid • Testosterone	• Caffeine, salicylic acid, and testosterone were simultaneously evaluated in a 6-well skin MPS. • Skin MPS exhibited a lower coefficient of variation than Franz diffusion cell, indicating improved precision and reproducibility. • Enabled parallel, high-throughput skin permeation screening, overcoming limitations of conventional Franz diffusion cells.
		Chaturvedi et al. [142]	(Cell-based) • Skin - Keratinocytes (HaCaT cell line) - Dermal Fibroblasts (primary)	Matrigel	• Caffeine • Salicylic acid • Hydrocorisone • Clotrimazole	• A Matrigel-based skin MPS showed functional barrier integrity, viscoelastic behavior, and differential permeation patterns for model drugs with different lipophilicities. • Enabled reference-based benchmarking of drug permeation behavior in a dynamic skin MPS, supporting its potential utility for preclinical transdermal studies.
		Zhang et al. [143]	(Cell-based) • Skin - Keratinocytes (primary)	None	• Naphthalene acetic acid • Isopropanol • Methyl stearate • Heptyl butyrate • Hexyl salicylate • Cyclamen aldehyde • 1-Bromohexane • Potassium hydroxide • 1-Methyl-3-phenyl-1-piperazine • Heptanal	• Non-irritant compounds such as isopropanol preserved barrier integrity, whereas irritant compounds including 1-bromohexane induced decreased TEER and increased paracellular permeation. • Enabled skin irritation assessment, allowing discrimination between irritant and non-irritant compounds using TEER and MTT analyses.
		Quan et al. [120]	(Cell-based) • Skin - Keratinocytes (HaCaT cell line) - Dermal Fibroblasts (primary)	Collagen type I	• *Propionibacterium acnes* • sodium lauryl sulfate(SLS) • dexamethasone • polyphyllin H	• In an inflammatory skin model induced by sodium lauryl sulfate (SLS) and *Propionibacterium acnes*, dexamethasone and polyphyllin H promoted barrier recovery and suppressed inflammatory responses. • Polyphyllin H exhibited superior barrier restoration and anti-inflammatory effects compared with dexamethasone after 24 h. • Enabled construction of an inflammatory skin model and comparative, time-dependent drug efficacy screening.
		Koning et al. [121]	(Cell-based) • Skin - Keratinocytes (primary) - Dermal Fibroblasts (primary) - Langerhans cells (MUTZ-3-derived) • Co-culture - gingiva keratinocytes (primary) - gingival fibroblasts (primary)	Collagen + Fibrin	• Nickel sulfate	• Topical application of nickel sulfate to the gingival compartment led to diffusion of nickel ions through the circulating medium into the skin, accompanied by Langerhans cell activation and migration toward the dermis, recapitulating immune sensitization. • Enabled reproduction of systemic immunotoxic pathways linking oral exposure to skin immune activation using an integrated oral–skin multi-organ MPS.
**Vascularized**	**Static**	Jusoh et al. [144]	(Cell-based) • Skin - Keratinocytes (primary) - Dermal Fibroblasts (primary) - Endothelial cells (primary)	Fibrin	• Sodium lauryl sulfate (SLS) • Steartrimonium chloride (SC)	• Exposure of the stratum corneum to irritants such as sodium lauryl sulfate (SLS) or steartrimonium chloride (SC) weakened keratinocyte tight junctions and enhanced endothelial sprouting, including increased sprout length, number, diameter, and lumen formation. • Enabled quantitative evaluation of irritation-induced angiogenesis by recapitulating epidermal barrier disruption and associated vascular responses within an angiogenesis-based skin MPS.
	**Dynamic**	Wufuer et al. [132]	(Cell-based) • Skin - Keratinocytes (HaCaT cell line) - Dermal Fibroblasts (HS27 cell line) - Endothelial cells (primary)	Porous membrane (Polyethylene terephthalate)	• Dexamethasone	• TNF-α perfusion into the dermal layer increased FITC-dextran permeability, consistent with enlarged paracellular gaps and an inflammatory/edematous state. • Topically applied dexamethasone diffused into the lower layers, suppressed inflammatory responses, and reduced vascular permeability. • Enabled quantitative assessment of inflammation–edema responses and transdermal anti-inflammatory efficacy in a three-layer epidermis–dermis–endothelium skin MPS.
		Mori et al. [131]	(Cell-based) • Skin - Keratinocytes (primary) - Dermal Fibroblasts (primary) - Endothelial cells (primary)	Collagen type I	• Caffeine • Isosorbide dinitrate (ISDN)	• Isosorbide dinitrate (ISDN) permeated more effectively than caffeine through the stratum corneum, and VEGF increased vascular uptake of both compounds. • Enabled *in vitro* quantification of lipophilicity-dependent transdermal transport and VEGF-induced increases in vascular permeability in a vascularized skin MPS.
		Salameh et al. [133]	(Cell-based) • Skin - Keratinocytes (primary) - Dermal Fibroblasts (primary) - Endothelial cells (primary)	Collagen type I	• Caffeine • Minoxidil	• Topical application of caffeine and minoxidil showed permeation profiles in the skin MPS consistent with those of native human skin. • Enabled concurrent evaluation of topical and systemic exposure and reproduction of barrier–vascular interactions governing transport of drugs and toxicants.
		Barros et al. [134]	(Cell-based) • Skin - Keratinocytes (primary) - Dermal Fibroblasts (primary) - Endothelial cells (primary) - Melanoma cells (B16F10 cell line)	Gelatin methacryloyl (GelMA) + alginate	• Doxorubicin	• A doxorubicin-loaded microneedle patch delivered a higher, more localized drug concentration to the tumor layer and achieved greater cytotoxicity than perfusion-channel (systemic-mimicking) delivery. • Enabled quantitative validation of epidermis-mediated localized delivery and evaluation of transdermal, tumor-targeted therapy using a skin cancer MPS.

**Table 3:** Summary of representative Franz diffusion cells and skin MPS studies compared with *in vivo* or conventional comparator data.

Model type	Subtype	Reference	Compound	Endpoint	Comparator
Conventional	Franz diffusion cell	Mohammed et al. [145]	Niacinamide	*In vitro* mean flux vs *in vivo* niacinamide signal intensity	Confocal Raman spectroscopy in human skin
	Franz diffusion cell	Simon et al. [146]	Rivastigmine	Fraction absorbed	Plasma PK
	Franz diffusion cell	Lukács et al. [139]	Caffeine	AUC	*In vivo* transdermal microdialysis
Skin MPS	Non-vascularized/Dynamic	Lukacs et al. [139]	Caffeine	AUC	*In vivo* transdermal microdialysis
	Non-vascularized/Dynamic	Alberti et al. [138]	Caffeine	Steady state flux (J_ss_), lag time, permeability coefficient (K_p_)	Franz diffusion cell
	Vascularized/Dynamic	Salameh et al. [133]	Caffeine, Minoxidil	Permeability coefficient (K_p_)	Franz diffusion cell

### 4.1 Non-vascularized skin MPS for performance assessment of transdermal drug delivery systems

#### 4.1.1 Non-vascularized static skin MPS

**Fig. 3:**
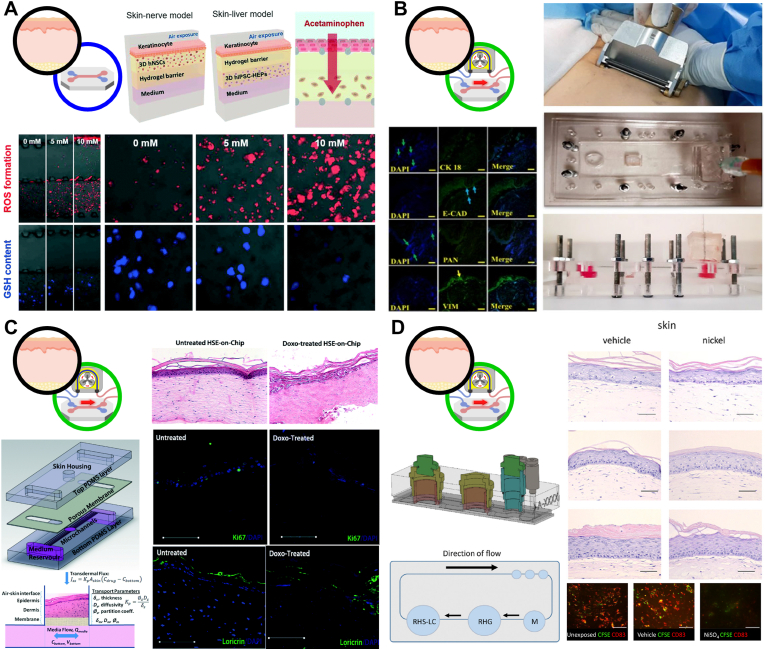
**Representative case studies of non-vascularized skin MPS for performance assessment of TDDS.** (A) A non-vascularized static skin MPS integrating the epidermal barrier with underlying neuronal or hepatic modules to quantify transdermal sensory irritation and hepatotoxicity. Reproduced with permission from Ref. [108]. Copyright © 2022, Royal Society of Chemistry. (B) A non-vascularized dynamic skin MPS based on *ex vivo* skin models to quantify retinoic acid permeation, including cumulative permeation and steady-state flux (J_ss_). Reproduced with permission from Ref. [91]. Copyright © 2023, Nature. (C) A non-vascularized dynamic skin MPS operated by gravity-driven flow to quantify transdermal permeability (K_p_) and drug-induced toxicity during long-term culture. Reproduced with permission from Ref. [107]. Copyright © 2015, Royal Society of Chemistry. (D) A non-vascularized dynamic oral-skin multi-organ MPS connecting reconstructed gingiva and reconstructed skin with Langerhans cells to model nickel ion transport and sensitization. Reproduced with permission from Ref. [121]. Copyright © 2022, Frontiers Media S.A.

Lee et al. developed a non-vascularized static skin MPS capable of assessing multi-organ toxicities induced by topically applied compounds, including sensory irritation and hepatotoxicity [108] (Fig. 3A). This platform integrated differentiated neural stem cell (NSC)- or induced pluripotent stem cell (iPSC)-derived neurons or hepatocytes beneath the epidermal layer, forming hybrid skin–nerve and skin–liver configurations. Sensory irritants such as capsaicin and lactic acid, along with the sensory-modulating compound strontium chloride, induced measurable neuronal activation quantified by real-time calcium imaging. In parallel, transdermal delivery of acetaminophen and camphor enabled assessment of hepatotoxicity via fluorescence-based quantification of reduced glutathione depletion and reactive oxygen species (ROS) generation. This study demonstrated that non-vascularized static skin MPS can functionally couple transdermal transport with distal organ-specific toxicity readouts.

#### 4.1.2 Non-vascularized dynamic skin MPS using *ex vivo* skin

Non-vascularized dynamic skin MPS based on *ex vivo* skin models have been extensively used for quantitative evaluation of transdermal permeation kinetics. Mohamadali et al. introduced a skin MPS incorporating *ex vivo* human skin mounted within a microfluidic chamber, where periodic medium replacement generated controlled flow beneath the tissue [91] (Fig. 3B). Using retinoic acid (RA) as a model lipophilic compound, cumulative permeation and steady-state flux (J_ss_) were quantified through time-resolved effluent analysis. Similarly, Lukács et al. employed an *ex vivo* skin MPS to compare caffeine permeation between intact and tape-stripped skin, demonstrating a marked increase in absorption following removal of the stratum corneum [139]. Compared with conventional Franz diffusion cells, this microfluidic platform offered a miniaturized dynamic setting with lower tissue and formulation requirements and continuous flow mimicking dermal circulation, while the *in vivo* microdialysis results showed the same overall trends in tape-stripping and species-dependent permeability as the *ex vivo* measurements. Bajza et al. further used this platform to reveal the role of P-glycoprotein (P-gp) in mediating transdermal absorption of quinidine and erythromycin, as P-gp inhibition significantly reduced permeation across *ex vivo* skin, Franz diffusion cells, and *in vivo* models [137]. Compared with the *in vivo* setting, where the same tendency was observed but was less pronounced in a multifactorial physiological environment, the skin MPS allowed clearer interrogation of P-gp–mediated transport under controlled *ex vivo* conditions. Disease-specific alterations in transdermal absorption were investigated by Szederkenyi et al., who constructed a dynamic skin MPS using *ex vivo* mouse skin representing healthy, psoriatic, and allergic dermatitis conditions [140]. Caffeine permeation increased substantially in disease-mimicking skin, reflecting impaired barrier function. Differential permeation kinetics of anti-inflammatory drugs (piroxicam, diclofenac, dexamethasone, and indomethacin) were further analyzed, revealing biphasic absorption behavior for dexamethasone and indomethacin. Integration of skin MPS data with a three-compartment *in silico* model successfully reproduced time-dependent permeation profiles across disease states, demonstrating the value of combining experimental and computational approaches for transdermal drug delivery prediction.

#### 4.1.3 Non-vascularized dynamic skin MPS using synthetic membranes and skin equivalents

Synthetic membrane-based and skin equivalent-based MPS provide scalable alternatives to *ex vivo* skin. De Mello et al. developed a recirculating body-on-a-chip system integrating cardiac and hepatic modules with a Strat-M synthetic membrane acting as a skin barrier [141]. Using various drugs including diclofenac, ketoconazole, hydrocortisone, and acetaminophen, a comparison between transdermal and systemic administration revealed substantially lower circulating drug concentrations under TDDS conditions, resulting in attenuated cardiac and hepatic toxicity. This platform highlighted the strong barrier effect of skin and the necessity of higher topical doses to achieve systemic exposure comparable to direct administration.

Bottom-up reconstructed skin MPS have demonstrated broader utility, ranging from long-term permeation testing to studies of barrier disruption, inflammation, and immunotoxicity. Abaci et al. developed a gravity-driven skin MPS using full-thickness human skin equivalents (HSE) that maintained stable epidermal and dermal structures for three weeks [107] (Fig. 3C). Permeation of a fluorescein-labeled oligonucleotide yielded a stable permeability coefficient (K_p_ ≈ 4 × 10^−3^ μm/s), while doxorubicin exposure induced dose-dependent cytotoxicity. Alberti et al. introduced a multi-unit skin MPS composed of six independent skin equivalents, enabling parallel permeation assays of three compounds (caffeine, salicylic acid, and testosterone) [138]. In direct comparison with Franz diffusion cells, this microfluidic platform also showed higher sensitivity in cumulative permeant measurements and comparable or lower coefficients of variation, likely owing to reduced analyte dilution and whole-perfusate analysis, thereby supporting more precise skin permeation testing. Chaturvedi et al. developed a Matrigel-based skin MPS and characterized its barrier integrity, mechanical properties, and drug permeation behavior using model compounds with different lipophilicities [142]. This skin MPS was validated against literature- and guideline-based reference benchmarks and showed drug permeability behavior consistent with accepted skin models, supporting its use as a preclinical dynamic alternative for transdermal studies. Zhang et al. developed a PET membrane-based skin MPS in which primary human keratinocytes differentiated into a multilayered epidermis [143]. Non-irritant compounds such as isopropanol preserved barrier integrity, whereas irritants including 1-bromohexane induced TEER reduction and increased paracellular permeability. Quan et al. applied SLS and *Propionibacterium acnes* to generate inflamed skin models, demonstrating that dexamethasone and polyphyllin H suppressed cytokine secretion and promoted barrier recovery, with polyphyllin H exhibiting superior efficacy [120]. Finally, Koning et al. extended the application of non-vascularized dynamic skin MPS to inter-organ immunotoxicity modeling by integrating reconstructed human gingiva and skin containing Langerhans cells within an oral–skin multi-organ MPS [121] (Fig. 3D). Oral exposure to nickel sulfate led to metal ion diffusion into the skin compartment, triggering Langerhans cell activation and migration, thereby recapitulating systemic sensitization pathways.

### 4.2 Vascularized skin MPS for performance assessment of transdermal drug delivery systems

#### 4.2.1 Vascularized static skin MPS

**Fig. 4:**
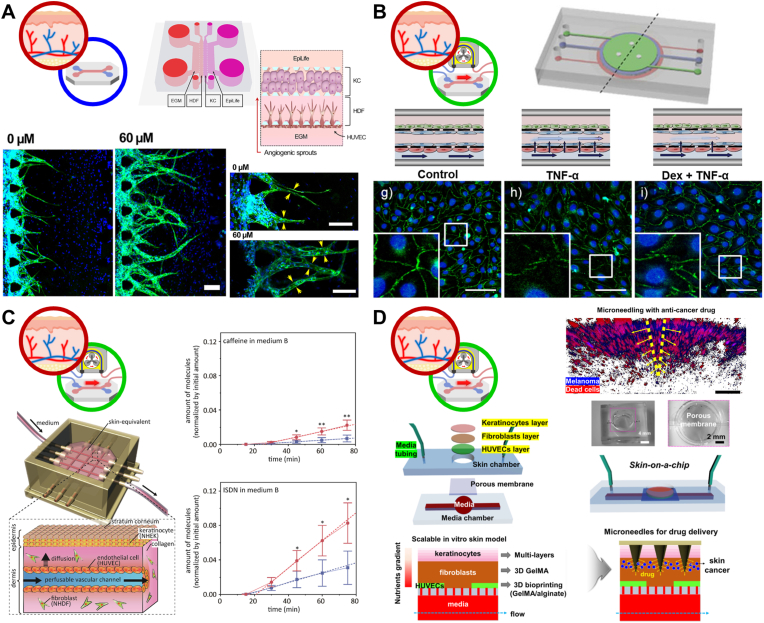
**Representative case studies of vascularized skin MPS for performance assessment of TDDS.** (A) A vascularized static skin MPS based on 3D angiogenesis to assess irritant-induced barrier disruption and vascular responses. Reproduced with permission from Ref. [144]. Copyright © 2019, AIP Publishing. (B) A vascularized dynamic skin MPS with membrane-separated compartments to model TNF-α-induced inflammation and quantify transdermal anti-inflammatory effects via vascular permeability. Reproduced with permission from Ref. [132]. Copyright © 2016, Nature. (C) A vascularized dynamic skin MPS featuring a nylon wire-templated intradermal vessel channel and an engineered stratum corneum to evaluate transdermal permeation of hydrophilic and lipophilic compounds. Reproduced with permission from Ref. [131]. Copyright © 2017, Elsevier. (D) A vascularized dynamic skin cancer invasion MPS with melanoma cells embedded in the dermis to compare topical GelMA microneedle-mediated delivery with perfusion-based delivery for tumor deposition and cytotoxic efficacy. Reproduced with permission from Ref. [134]. Copyright © 2025, Elsevier.

As a representative application of a vascularized static skin MPS, Jusoh et al. developed a skin irritation testing platform incorporating 3D angiogenesis to quantitatively evaluate vascular responses to topical irritant [144] (Fig. 4A). SLS and steartrimonium chloride (SC) were applied to the stratum corneum, and subsequent changes in epidermal tight junction integrity and angiogenic microvascular morphology were analyzed. Quantitative metrics included branch number, lumen formation, and endothelial sprouting. Irritant treatment led to disruption of keratinocyte tight junctions and increased angiogenic activity, demonstrating that the platform could simultaneously capture epidermal barrier damage and corresponding vascular responses. This study highlighted the utility of static vascularized skin MPS for quantifying irritation-induced angiogenesis in the absence of flow.

#### 4.2.2 Vascularized dynamic skin MPS

Vascularized dynamic skin MPS further enhance physiological relevance by incorporating perfusion-mediated transport and endothelial mechanobiology. Wufuer et al. developed a three-layer skin MPS comprising epidermal, dermal, and endothelial compartments separated by porous membranes [132] (Fig. 4B). Controlled perfusion of tumor necrosis factor-α (TNF-α) into the dermal layer induced an inflammatory state. Vascular permeability was quantified using a FITC–dextran assay, revealing increased paracellular transport due to inflammation-induced junctional disruption. Dexamethasone applied topically to the epidermal layer diffused into the lower compartments, effectively suppressing inflammatory responses and restoring vascular barrier integrity. This study demonstrated that vascularized dynamic skin MPS enable quantitative evaluation of inflammation-associated edema and therapeutic efficacy during transdermal delivery. More advanced vascularized skin MPS incorporate perfusable luminal structures within the dermal layer to enable direct sampling of vascular effluents. Mori et al. developed a skin MPS in which a vascular channel was formed using a nylon wire template, while a stratified stratum corneum was reconstructed in the epidermal layer [131] (Fig. 4C). Permeation of hydrophilic caffeine and lipophilic isosorbide dinitrate (ISDN) was quantified in both medium from vascular channel and medium under skin-equivalent. ISDN exhibited higher permeation efficiency than caffeine, consistent with the lipid-rich nature of the stratum corneum. Introduction of vascular endothelial growth factor (VEGF) into the vascular channel increased endothelial permeability, resulting in enhanced transport of both compounds. This response reflected physiologically relevant modulation of vascular barrier function. In a similarly constructed model, Salameh et al. developed a vascularized skin MPS integrating epidermal and dermal layers with three perfusable vascular channels interconnected by a vasculogenesis-driven microvascular network [133]. Topical application of caffeine and minoxidil enabled determination of permeation coefficients that closely matched those reported for native human skin. Notably, perfused models exhibited improved epidermal barrier maturation and reduced overall permeability compared with non-perfused controls. In addition, polycyclic aromatic hydrocarbons (PAHs), a well-known group of environmental pollutants, when introduced through the vascular channels, showed enhanced delivery into the skin tissue, confirming that organized vascular architecture facilitates bidirectional compound transport between skin and circulation. Beyond conventional *ex vivo* diffusion assays that primarily assess passive permeation, this vascularized skin MPS offered the structural advantage of enabling simultaneous evaluation of topical delivery and downstream vascular or systemic exposure, consistent with the authors’ conclusion that perfusion and a complex vascular plexus improve the predictive value of the model.

#### 4.2.3 Advanced applications: microneedle-enabled drug delivery

Vascularized skin MPS have also been applied to evaluate advanced TDDS strategies in disease contexts. Among advanced TDDS strategies, microneedles represent a prototypical physical enhancement approach. Microneedles are arrays of micron-scale projections (typically ∼0.1–1 mm in length) that transiently perforate the stratum corneum to create aqueous microchannels, thereby enabling efficient transdermal delivery of therapeutics that are otherwise poorly permeable, including hydrophilic compounds and macromolecules. Barros et al. developed a skin cancer invasion model by embedding melanoma cells within the dermal layer of a vascularized skin MPS [134] (Fig. 4D). A gelatin methacryloyl (GelMA) microneedle patch loaded with doxorubicin was applied to the skin surface to achieve localized drug delivery. This approach was compared with systemic delivery via perfusion through the vascular channel. Microneedle-mediated delivery resulted in higher local drug accumulation within the tumor region and significantly greater cytotoxic efficacy than perfusion-based administration, demonstrating the advantage of spatially targeted transdermal delivery in vascularized skin models.

## 5 Perspective

Although skin MPS have substantially advanced TDDS research, important challenges remain in reproducing the full structural and physiological complexity of human skin and in improving reproducibility, standardization, and translational utility [95]. The following sections outline major future directions for the field.

### 5.1 Modeling the complex structural architecture of human skin

**Fig. 5:**
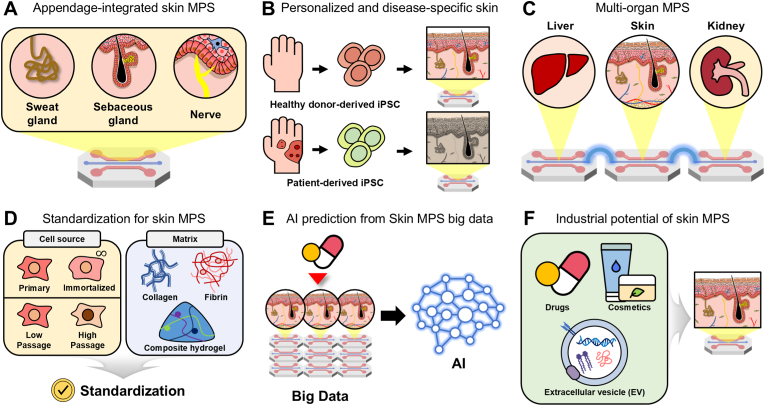
**Perspectives and future directions of skin MPS for TDDS.** (A) Modeling the complex structural architecture of human skin through appendage-integrated skin MPS (sweat gland, sebaceous gland, and nerve). (B) Stem cell-based personalized and disease-specific skin modeling using healthy donor- and patient-derived iPSC. (C) Extending skin MPS toward multi-organ MPS by connecting skin modules with systemic organs such as liver and kidney. (D) Standardization for skin MPS in TDDS, highlighting key criteria for cell sources and matrix/hydrogel selection. (E) Big-data generation from sensor-integrated skin MPS to enable AI-based prediction. (F) Industrial applications of skin MPS in TDDS across pharmaceuticals, cosmetics, and extracellular vesicle (EV) delivery.

Although significant progress has been achieved in applying MPS technologies to TDDS, several technical limitations remain unresolved. Among these, improving reproducibility is a critical requirement for skin MPS to reliably predict human skin responses (Fig. 5A). Many existing platforms rely on simplified or partially reconstructed skin models that exhibit limited structural and compositional similarity to native tissue [87]. To improve predictive accuracy, next-generation skin MPS should more precisely reconstruct the skin microenvironment, including appendages such as sweat glands, sebaceous glands, and hair follicles [97,111]. Because these structures contribute to permeation, local drug accumulation, metabolism, and immune regulation, their incorporation would broaden the utility of skin MPS for TDDS as well as for toxicity, inflammation, and aging studies [147,148].

### 5.2 Stem cell–based personalized and disease-specific skin modeling

iPSC, generated through reprogramming of patient-derived somatic cells, can differentiate into key skin cell types such as keratinocytes, fibroblasts, and melanocytes [[149], [150], [151]]. This technology has attracted growing interest in skin MPS development because a single iPSC line enables scalable production of genetically uniform skin cells (Fig. 5B). Through intrinsic self-organization processes, iPSC-derived cells can form 3D skin organoids in which epidermal and dermal compartments are spatially organized. More complex skin-mimetic structures incorporating hair follicles, sebaceous glands, melanocytes, and neural components have also been reported [[152], [153], [154]].

Integration of these organoids or engineered 3D skin tissues into MPS enables the construction of iPSC-based personalized skin MPS composed of cells sharing an identical genetic background [155,156]. This approach minimizes genetic heterogeneity among cellular components and allows systematic evaluation of inter-patient variability in drug response and skin permeability. Moreover, the use of autologous cell sources permits repeated generation of identical tissue constructs, reducing inter-experimental variability and improving data reproducibility [155,157].

Beyond personalization, iPSC-based skin MPS are valuable for modeling genetic skin disorders and rare diseases. Patient-derived iPSCs retain disease-causing mutations and can reproduce pathological features such as junctional defects, basement membrane instability, and abnormal keratin organization [[158], [159], [160]]. In addition, CRISPR/Cas9-mediated correction of the same iPSC lines enables direct comparison of tissue structure and barrier function before and after editing, providing a platform for evaluating gene correction strategies [[161], [162], [163]]. Together, these models support studies of disease mechanisms, drug responsiveness, and personalized therapeutic development [164].

### 5.3 Extending skin MPS toward multi-organ MPS

Skin MPS are effective platforms for modeling localized transdermal absorption and barrier responses. However, standalone skin models are inherently limited in reproducing systemic PBPKs and post-absorption metabolism [165,166]. To address this gap, multi-organ MPS have been developed by interconnecting skin modules with other major organs through microfluidic networks that emulate systemic circulation [109,[167], [168], [169]] (Fig. 5C).

Representative systems such as skin–liver–kidney MPS enable *in vitro* reconstruction of key ADME processes, including systemic uptake, hepatic metabolism, and renal excretion, thereby improving prediction of systemic exposure and toxicokinetic behavior [[170], [171], [172], [173], [174]]. Multi-organ platforms also expand TDDS research beyond pharmacokinetics by enabling analysis of immune and metabolic crosstalk. For example, skin–gut MPS can provide mechanistic insight into bidirectional interactions between transdermally absorbed compounds and gut-derived responses relevant to inflammatory skin disorders [[175], [176], [177], [178], [179]].

More complex configurations such as skin–liver–heart MPS have been applied to assess systemic toxicity, organ–organ interactions, and metabolite accumulation following drug exposure [141,180]. Such platforms are increasingly used to study drug–drug interactions and chronopharmacology, as they can capture circulation-dependent exposure dynamics *in vitro* [181]. Notably, emerging evidence indicates that skin functions as an active regulator of systemic physiology. Senescence-associated secretory phenotype (SASP) factors released by aged skin cells can impair distant organ function, challenging the traditional view of skin as merely a passive barrier [182].

Future multi-organ MPS are expected to incorporate aged or disease-specific skin modules, enabling investigation of how transdermal exposure and skin-derived pathological signals influence distant organs in systemic drug response and age-related disease contexts [183,184].

### 5.4 Standardization for skin MPS in transdermal drug delivery systems

Standardization of cell sources and matrix composition is essential for applying skin MPS to regulatory assessment of TDDS (Fig. 5D). Because skin MPS may incorporate keratinocytes, fibroblasts, endothelial cells, and immune cells, variation in cell origin, passage number, and differentiation state can strongly affect barrier function and model reproducibility. For example, primary keratinocytes generally achieve better stratification and tighter barrier formation, whereas HaCaT cells are more practical for long-term culture and mechanistic studies despite their lower differentiation fidelity [[185], [186], [187]]. These differences highlight the need for application-specific criteria in cell source selection.

Matrix composition is another key determinant of skin MPS performance and physiological relevance [188]. Commonly used matrices include type I collagen and fibrin [97,135,144,189]. Type I collagen supports fibroblast interactions and keratinocyte differentiation but suffers from severe contraction when used alone [190]. Fibrin is advantageous for vascularization but more closely resembles a wound-healing environment than healthy dermis, limiting its suitability for stable long-term skin models [191]. To address these limitations, recent efforts have focused on decellularized extracellular matrix (dECM) and composite hydrogels. Skin-derived dECM retains native biochemical cues and three-dimensional architecture, enhancing physiological responsiveness and improving tissue reproducibility when incorporated into skin MPS [135,192,193]. Composite hydrogels combining natural or synthetic polymers—such as hyaluronic acid (HA) and GelMA—reduce collagen contraction and allow precise tuning of mechanical stiffness via photocrosslinking [194,195]. These features improve structural stability and increase the feasibility of standardizing MPS platforms across laboratories.

#### 5.4.1 Regulatory alignment and standardization of skin MPS readouts

Skin MPS must move beyond model-specific performance claims toward standardized validation frameworks aligned with existing regulatory paradigms. Recent reviews suggest that, for skin MPS to be applied in actual nonclinical and cosmetic safety evaluation, they should be established within a standardized validation framework that can be interpreted in alignment with the logic of current OECD test guidelines and the direction of FDA modernization [196]. Consistent with this view, this section maps TEER, flux-based permeation, cumulative permeation, and recovery onto the regulatory logic of OECD TG 428 and FDA *in vitro* permeation test (IVPT) (Table 4). OECD TG 428 provides a regulatory framework for *in vitro* skin absorption testing using excised human or animal skin mounted in static or flow-through diffusion cells [76]. OECD TG 428 includes barrier integrity, flux-based permeation, cumulative permeation, and recovery as evaluation-related parameters. However, for most of these parameters except recovery, the guideline places greater emphasis on how the study should be conducted and how the data should be reported, rather than providing universal numerical cutoffs. Specifically, barrier integrity requires confirmation of skin integrity prior to testing and demonstration that barrier function has been maintained when stored skin is used, whereas flux-based permeation and cumulative permeation are evaluated through time-dependent analysis of receptor fluid, presentation of the absorption profile, and interpretation of the total absorbed amount. In contrast, recovery is explicitly defined as a mass balance parameter across the entire test system, for which a more concrete target criterion is provided, namely a mean of 100 ± 10%. Similarly, the FDA IVPT guidance treats barrier integrity, flux-based permeation, cumulative permeation, and recovery as important evaluation-related parameters, but it does not apply the same type of acceptance criterion to each of them [197]. For barrier integrity, although no universal single cutoff is provided, a method- and instrument-specific acceptance criterion should be predefined and justified, specified in the protocol, and reported for each skin section. In contrast, no universal cutoff in the strict sense is provided for flux-based permeation or cumulative permeation. Instead, the guidance emphasizes that sufficiently informative flux and cumulative permeation profiles should be generated and reported using an appropriate study duration and sampling schedule. In this context, flux is represented by J_max_ and cumulative permeation by AMT, both of which are used as bioequivalence endpoints in the pivotal study. By contrast, recovery is not treated as a whole-system mass balance criterion as in OECD TG 428, but rather as a reporting metric used to estimate dose depletion based on the amount of drug recovered in the receptor solution. Taken together, these regulatory frameworks suggest that standardization of skin MPS should focus not only on numerical performance, but also on clarifying whether each readout functions as a barrier qualification criterion, a permeation-based bioequivalence endpoint, or a mass balance and reporting parameter within a regulatory context.

**Table 4:** Regulatory interpretation of representative skin MPS readouts according to OECD TG 428 and FDA IVPT guidance [76,197].

MPS readout	Measured feature	OECD TG 428	FDA IVPT
**Barrier integrity**	Electrical barrier integrity of the epidermal layer	• Maintenance of barrier function should be demonstrated for stored skin. • No universal cutoff is provided.	• Method-specific acceptance criterion required. • Acceptable approaches may include Trans-epidermal water loss (TEWL), tritiated water, or electrical based skin barrier integrity test (TEER).
**Flux-based permeation (**J_ss_/J_max_**)**	Rate of drug permeation across the skin model	• Time-course receptor fluid analysis and rate-based data reporting required. • No universal cutoff is provided.	• Indicidual cell-based flux calculation and log-transformed the maximum flux (J_max_) evaluation required. • No universal cutoff is provided.
**Cumulative permeation (AMT)**	Total amount of drug transported into the receptor compartment over the duration	• Time-dependent absorption profile and total absorbed amount should be reported • No universal cutoff is provided.	• Evaluation of log-transformed AMT as a pivotal endpoint for bioequivalence required. • No universal cutoff is provided.
**Recovery**	Mass balance across all compartments (OECD TG 428)/Receptor recovery for dose depletion assessment (FDA IVPT)	• Analysis of all compartments and mass balance assessment required. • Target mean 100 ± 10%.	• Receptor recovery should be reported as dose depletion. • No acceptance criterion is provided.

### 5.5 Big data generation from sensor-integrated skin MPS for artificial intelligence-based prediction

Recent advances have integrated impedance-based TEER electrodes, electrochemical sensors, and optical or fluorescent sensors into skin MPS, enabling real-time monitoring of barrier integrity, oxygen concentration, pH, and metabolite dynamics [[198], [199], [200]] (Fig. 5E). The high-frequency time-series datasets provide essential big data resources for artificial intelligence (AI) training and enable quantitative analysis of temporal cellular responses and drug permeation kinetics [201,202]. Simultaneous acquisition of sensor signals and experimental metadata—such as flow rate, duration of ALI culture, and channel geometry—improves reproducibility, facilitates data standardization, and supports experimental automation and high-throughput screening [85,[203], [204], [205]]. Recent studies further demonstrate that sensor-integrated skin-interfacing platforms are evolving toward continuous monitoring systems capable of generating complex physiological datasets, and that AI-assisted transdermal platforms may leverage such data for predictive modeling and adaptive drug delivery [206,207].

AI models trained on these datasets enable prediction of drug permeability (K_p_), steady-state flux (J_ss_), and barrier recovery metrics, as well as optimization of experimental conditions with skin MPS [[208], [209], [210], [211], [212]]. Machine learning approaches, which rely on predefined features, are effective for incorporating quantitative variables such as TEER, flow rate, skin thickness, and compound physicochemical properties [209,213]. In contrast, deep learning models based on artificial neural networks (ANNs) can autonomously extract features from high-dimensional inputs, including TEER time-series data, fluorescence signals, and imaging data, to predict drug–skin interactions and optimize system parameters [209,[214], [215], [216]]. These AI-driven predictions can be extended to *in silico* modeling, enabling computational simulation of *in vitro* or *in vivo* outcomes [217,218]. In particular, inverse formulation design frameworks allow target performance metrics—such as permeation efficiency or barrier recovery—to be specified, after which optimal physicochemical and formulation parameters are back-calculated [219,220]. Integration of skin MPS-derived big data with PBPK models further enhance IVIVE accuracy [221,222]. From a practical perspective, AI-integrated skin MPS improve both design efficiency and reproducibility. Learning from sensor-derived data reduces the need for exhaustive empirical testing and enables rational selection of MPS architectures and flow conditions based on predefined performance criteria [223,224]. At the same time, AI-based normalization of sensor outputs minimizes noise and inter-experimental variability, ensuring consistent data generation across studies [215,225]. Together, these capabilities establish a foundation for standardized, predictive skin MPS platforms [223,226].

### 5.6 Industrial potential of skin MPS in advancing transdermal drug delivery systems

Skin MPS are expected to drive a paradigm shift in TDDS research across pharmaceutical and cosmetic industries (Fig. 5F). In pharmaceutical development, these platforms enable rapid, high-throughput evaluation of transdermal absorption efficiency across diverse compounds and formulations [200]. They also provide alternative approaches for bioequivalence assessment in generic drug development and enable prediction of inter-individual variability using patient-derived skin MPS [[227], [228], [229]]. In the cosmetic industry, skin MPS are increasingly adopted as preclinical platforms to replace animal testing for skin irritation, anti-aging efficacy, and phototoxicity assessment [95]. Recent anti-aging research has increasingly focused on extracellular vesicles (EVs), including exosomes, for which transdermal delivery is preferred due to regulatory limitations on invasive administration [230]. Delivery systems such as microneedles, liposomes, and nanoemulsions have been developed to enhance EV skin penetration, and skin MPS offer a quantitative and reproducible means to validate their effectiveness [231,232]. Furthermore, the establishment of standardized validation protocols and integration with *in silico* prediction models are expected to accelerate industrial adoption of skin MPS. These advances support large-scale screening and quantitative TDDS evaluation and increase the likelihood of regulatory recognition of skin MPS as alternative testing methodologies [233]. Such recognition would streamline preclinical development and reduce barriers to clinical translation.

#### 5.6.1 Quantitative translation roadmap: from skin MPS flux to systemic exposure

**Fig. 6:**
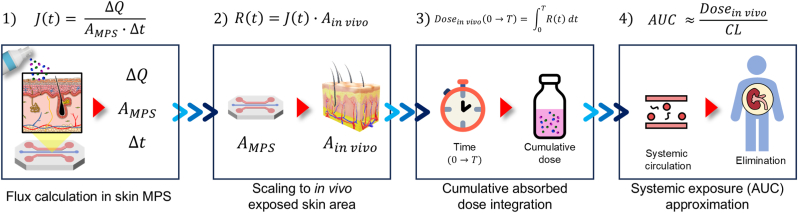
**Workflow for translating skin MPS permeation readouts into systemic exposure.** Time-resolved flux measured in the skin MPS (J(t)) is scaled to the intended *in vivo* exposed skin area ($A_{in\;vivo}$) to estimate the systemic input rate (R(t)), which is then integrated over time to calculate the cumulative absorbed dose (${Dose}_{in\;vivo}\left(0\to T\right)$) and approximate systemic exposure (AUC) under a simplified linear pharmacokinetic assumption.

To support the industrial application of skin MPS in TDDS development, their experimental outputs must be translated beyond comparative *in vitro* readouts into parameters that are relevant to *in vivo* drug exposure [234] (Fig. 6). Although skin MPS can provide time-resolved readouts of drug transport across a defined skin construct, these measurements are inherently normalized to the local device area and therefore cannot be directly interpreted as whole-body exposure. To address this gap, MPS-derived permeation data can be reformulated as an absorbed input function and subsequently linked to systemic pharmacokinetic parameters [235]. Skin MPS experiments can directly provide the permeated amount during a sampling interval (ΔQ), steady-state flux (J_ss_), permeability coefficient (K_p_), and total cumulative amount (AMT) [197]. In the scaling equations below, $A_{MPS}$ represents the effective permeation area of the skin MPS, whereas $A_{in\;vivo}$ represents the corresponding exposed skin area *in vivo* [236]. In a previous transdermal modeling study, systemic input was formulated as a function of area-normalized flux and patch surface area [236]. In the present framework, this area-based scaling concept was extended to skin MPS by replacing patch area with the intended *in vivo* exposed skin area. R(t) is the systemic input rate, ${Dose}_{in\;vivo}\left(0\to T\right)$ is the cumulative systemically absorbed dose over time, AUC is the systemic area under the concentration-time curve, and systemic clearance (CL) is the pharmacokinetic parameter used to link absorbed dose to systemic exposure [237]. The following simplified scaling scheme outlines how flux- and permeation-based outputs from skin MPS may be converted into estimates of absorbed dose and AUC [197,236,237].

1) The time-resolved flux from the skin MPS is calculated as follows [197]:

$$J\left(t\right)=\frac{\Delta Q}{A_{MPS}\cdot \Delta t}$$

where J(t) is the flux during sampling interval.

2) The systemic input rate is estimated by scaling flux to the intended *in vivo* exposed skin area [236]:

$$R\left(t\right)=J\left(t\right)\cdot A_{in\;vivo}$$

3) The total systemically absorbed dose is obtained by integrating the absorbed input rate over time [237]:

$${Dose}_{in\;vivo}\left(0\to T\right)=\int_{0}^{T}R\left(t\right)\;dt$$

4) If cumulative permeation is available as AMT, the systemically absorbed dose can be estimated more simply as [197,237]:

$${Dose}_{in\;vivo}\left(0\to T\right)=AMT\left(T\right)\cdot A_{in\;vivo}$$

5) Under a simplified linear pharmacokinetic assumption, systemic exposure can then be approximated as [237]:

$$AUC\approx \frac{{Dose}_{in\;vivo}}{CL}$$

Because CL reflects whole-body elimination rather than skin transport, it should be obtained from independent pharmacokinetic sources rather than from the skin MPS itself. Therefore, skin MPS-derived flux informs the absorption component of IVIVE, while prediction of systemic AUC requires coupling this input with an externally defined clearance term [238]. Taken together, this simplified framework provides a practical basis for translating *in vitro* skin permeation data into quantitative estimates of systemic exposure.

## 6 Conclusion

Skin MPS represent a transformative approach for addressing the physiological limitations of conventional *in vitro* models used in transdermal drug delivery research. As highlighted throughout this review, their central strength lies in the ability to tailor model complexity to the intended application, ranging from non-vascularized static systems for high-throughput barrier screening to vascularized dynamic systems capable of supporting PBPK analysis and complex toxicity evaluation. Continued advancement of skin MPS will require improved reconstruction of native skin architecture, including hair follicles and sebaceous glands, and immune components, together with functional maturation of patient-derived models. Equally important is the evolution of skin MPS toward quantitative predictive platforms through integration of *in vitro* transport data with PBPK modeling frameworks via IVIVE strategies. Achieving this goal will depend on the adoption of sensor-integrated, AI-assisted automation, rigorous standardization of cell and matrix components, and systematic cross-validation in collaboration with regulatory agencies. Collectively, these developments position skin MPS as a promising foundation for human-relevant, animal-free evaluation of TDDS and their eventual acceptance as regulatory-grade testing platforms.

## CRediT authorship contribution statement

**Jongwoo Ahn:** Conceptualization, Investigation, Validation, Visualization, Writing – original draft. **Geonho Jin:** Conceptualization, Investigation, Validation, Visualization, Writing – original draft. **Minji Cho:** Visualization. **Minho Do:** Investigation. **Jungho Ahn:** Validation. **Jihoon Ko:** Funding acquisition, Supervision, Writing – review & editing. **Seokyoung Bang:** Conceptualization, Funding acquisition, Supervision, Validation, Writing – review & editing.

## Declaration of competing interest

The authors declare no conflict of interest in this work.

## Data Availability

No data was used for the research described in the article.
